# Selective ablation of striatal striosomes produces the deregulation of dopamine nigrostriatal pathway

**DOI:** 10.1371/journal.pone.0203135

**Published:** 2018-08-29

**Authors:** Kirill Shumilov, M. Ángeles Real, Alejandra Valderrama-Carvajal, Alicia Rivera

**Affiliations:** Department of Cell Biology, Universidad de Málaga, Instituto de Investigación Biomédica, Málaga, Spain; Queens College, UNITED STATES

## Abstract

The striatum is a complex structure in which the organization in two compartments (striosomes and matrix) have been defined by their neurochemical profile and their input-output connections. The striosomes receive afferences from the limbic brain areas and send projections to the dopamine neurons of the substantia nigra pars compacta. Thereby, it has been suggested that the striosomes exert a limbic control over the motor function mediated by the surrounding matrix. However, the functionality of the striosomes are not completely understood. To elucidate the role of the striosomes on the regulation of the nigral dopamine neurons, we have induced specific ablation of this compartment by striatal injections of the neurotoxin dermorphin-saporin (DS) and dopamine neurotransmission markers have been analyzed by immunohistochemistry. The degeneration of the striosomes resulted in a nigrostriatal projections imbalance between the two striatal compartments, with an increase of the dopamine neurotransmission in the striosomes and a decrease in the matrix. The present results highlight the key function of the striosomes for the maintenance of the striatal dopamine tone and would contribute to the understanding of their involvement in some neurological disorders such as Huntington’s disease.

## Introduction

The caudate putamen (CPu) is one of the main nuclei of the basal ganglia which engages a variety of functions like the control of voluntary movement, learning from habit formation to complex motor sequences, decision-making and motivational behavior [1–3]. The CPu receives glutamatergic afferents from different cortical areas, the thalamus and the amygdala, as well as dopaminergic inputs from the substantia nigra pars compacta (SNc) [4]. Two primary striatofugal projection pathways have been established according to their projection targets: i) direct pathway, which innervates the substantia nigra pars reticulata (SNr); and ii) indirect pathway which also provides efferents to the SNr, through relay connections to globus pallidus (GP) and subthalamic nucleus (STh) [4]. The most abundant striatal neurons (90%) consist of medium-sized spiny projecting neurons (MSNs) which use GABA as neurotransmitter. The remaining striatal neurons (10%) are interneurons that have been classified into four populations according to their neurochemical profile [5]: i) cholinergic neurons (ChAT); ii) GABA interneurons that express somatostatin (SS), neuropeptide Y (NPY) and nitric oxide synthase (NOS); iii) GABA interneurons that contain parvalbumin (PV); and iv) GABA interneurons that express calretinin (CR).

Despite the noticeable homogeneous distribution of the striatal neurons, the expression of a range of neurochemical markers and the specificity of incoming and outcoming projections in different striatal subregions disclose a rather complex organization of the CPu [6–8]. Firstly, three striatal domains have been defined along the dorsolateral to the ventromedial axis based on their afferent connections, which have been called sensorimotor, associative and limbic domains [8]. Secondly, two compartments, embedded within this topographic organization, have been described: the striosome and matrix [6,7,9,10]. The striosomal compartment (~15% striatal volume) is a labyrinthine structure with connections predominantly coming from limbic areas, whereas the surrounded matrix is mainly related to sensorimotor and associative regions [7,11]. More recent studies have demonstrated that the striosomes provide direct projections to dopamine neurons of the SNc [12,13], which in turn project back to the CPu. In addition to these different connectivity features, the differential expression of a great variety of neurotransmitter-related signals has been extensively described in both compartments [7]. Among them, the μ opioid receptor (MOR) is especially enriched in the striosomes and has been used as a marker for these structures [14,15].

Nowadays, the functions of the striosome are not completely understood. Nevertheless, several studies have revealed an important role of this compartment in opiate reward-driven behaviors [16], decision-making and motor learning tasks [17], and behavioral adaptation [18]. Besides, the imbalance between striosome-matrix function has been related to several neurologic disorders, e.g. Huntington’s disease, drug addiction or Parkinson’s disease [7].

One of the methods employed to study striosomal function has been the selective ablation of neurons expressing MOR by using a dermorphin-saporin toxin (DS). Dermorphin is a high selective MOR agonist which induces receptor internalization [19], whereas saporin is a toxin that yields the inactivation of the ribosomes [20]. Therefore, the internalization of DS produces the degeneration of neurons expressing MOR and their efferent projections [21], while non-MOR neurons remain unaffected. This method has successfully been used to induce the ablation of both MOR enriched medullary neurons [22] and striosomal MSNs of the CPu [21,23–25].

The main objective of this study was to increase our knowledge in the role of the striosomal projection onto the dopamine neurons of the SNc and its impact on the nigrostriatal dopamine pathway. Furthermore, the effect of DS lesion on striatal interneurons have also been determined. To address this question, we have improved the lesion of the striosomes with DS, in order to obtain a higher degree of striosomal ablation in the whole CPu.

## Material and methods

### Animals

Male Sprague Dawley rats (n = 12) (Charles River, Barcelona, Spain) weighing 150–250 g were maintained on a standard light/dark cycle (12/12 h) and constant room temperature (20 ± 2°C). The rats had free access to food pellets and filtered water. Animal care and use followed guidelines from the European Union Council Directive (2010/63/EU) as well as the Spanish Government (Real Decreto 53/2013) and the experimental procedure was approved by the Ethical Committee of the University of Málaga (CEUMA 2012-0017-A). All efforts were made to minimize animal suffering and to reduce the number of animals used.

### Intrastriatal injections

**Fig 1:**
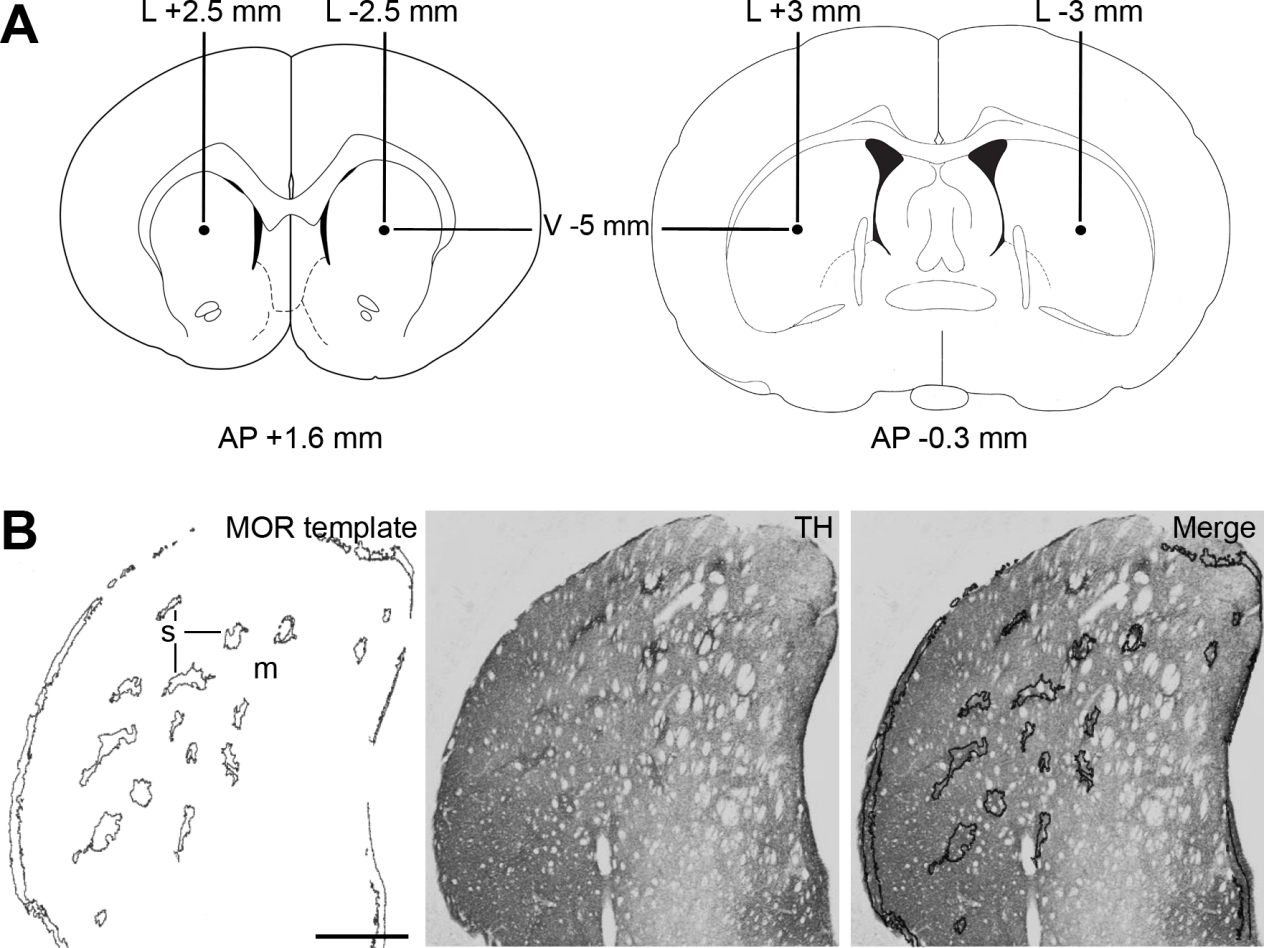
Schematic representation of procedures. (**A**) Diagrams of coronal brain plates modified from a rat brain atlas [26] depicting the sites where the stereotaxic injections were made. (**B**) Template obtained from a MOR immunolabeled brain section showing the distribution of the striosomes (s) into the dorsal striatum. The template is applied over the photomicrography of a section immunolabeled for TH, DAT or VMAT-2 (in this figure illustrated by TH) to quantify the optical density of each marker in the striosomal (s) and matrix (m) compartments. Abbreviations: AP: anteroposterior; L: lateral; V: ventral. Scale bar is 1 mm.

Unilateral intrastriatal injections of dermorphin-saporin conjugate (DS) (Advanced Targeting System, San Diego, CA, USA) were performed to induce the selective ablation of the striosomal compartment. To ensure the highest degree of injury, the infusion of DS was made at two different levels along the rostro-caudal axis of the CPu. The contralateral hemisphere was injected at the same conditions with 0.9% NaCl (vehicle) and it was used as control. Animals (n = 9) were deeply anaesthetized with ketamine (75 mg/kg, i.p.) and medetomidine (0.5 mg/kg, i.p.) and placed in a stereotaxic frame (Panlab, Barcelona, Spain). Two drills in each hemisphere were carried out to open the skull at the following coordinates from Bregma (in mm): i) AP = +1.6, L = ±2.5, V = -5; ii) AP = -0.3, L = ±3.0, V = -5 (Fig 1A) [26]. The infusion of 2 μl of either DS (17 μg/μl in saline) or vehicle per site was made using a 26-gauge Hamilton microsyringe (Hamilton®, Bonadunz, Switzerland) at a constant flow of 0.5 μl/min. Control animals (n = 3) received injections of unconjugated saporin (SAP, 17 μg/μl) (Advanced Targeting System, San Diego, CA, USA) and vehicle at the same striatal levels as those previously described. After injections, the skin was sutured and a topical antiseptic was applied. During the surgery and recovery, animals were kept warm using a heating pad and they received the appropriate post-operative care.

### Tissue preparation

Eight days after intrastriatal injections, rats were deeply anesthetized with sodium pentobarbital (60 mg/kg, i.p.) and perfused transcardially with 0.1 M phosphate-buffered saline, pH 7.4 (PBS) followed by 4% paraformaldehyde (w/v) in PBS. The brains were removed, post-fixed in the same fixative (overnight), cryoprotected with 30% sucrose diluted in PBS with 0.02% sodium azide (72 h) and frozen in dry ice. Rostro-caudal series of coronal sections (30 μm thick) were obtained with a freezing microtome (CM 1325, Leica, Weztlar, Germany) and stored at 4°C in PBS containing 0.02% sodium azide until use.

### Single immunohistochemical labeling

Free-floating sections were processed for standard single immunohistochemical labeling as previously described [27]. The sections were incubated for 24–48 h at RT with one of the following primary antibodies: rabbit polyclonal anti-μ opiod receptor (MOR, 1:50,000); mouse monoclonal anti-tyroxine hydroxylase (TH, 1:1,000); rabbit polyclonal anti-dopamine transporter (DAT, 1:1,000); or rabbit polyclonal anti-vesicular monamine transporter 2 (VMAT-2, 1:1,000) (Table 1). The primary antibodies were diluted in PBS with 0.2% Triton X-100 (PBS-TX) and 0.1% sodium azide.

**Table 1:** Primary antibodies employed in this study.

Antibody	Type	Specie	Reference (catalog #)	Dilution
**Calretinin**	Poly-	G	Swant (CG1)	1:10,000
**Choline acetyltransferase**	Poly-	G	Millipore (AB144P)	1:750/1:500^a^
**Dopamine transporter**	Poly-	R	Millipore (AB1591P)	1:1,000
**μ opioid receptor**	Poly-	R	Millipore (PC165L-100UL)	1:50,000/1:2000^a^
**Parvalbumin**	Mono-	M	Sigma-Aldrich (P3171)	1:5,000
**Somatostatin**	Poly-	G	Santa Cruz (sc-7819)	1:5,000
**Tyrosine hydroxylase**	Mono-	M	InmunoStar (P22941)	1:1,000
**Vesicular monoamine transporter 2**	Poly-	R	Millipore (AB1598P)	1:1,000

Abbreviations: Mono-, monoclonal; Poly-, polyclonal; G, goat; M, mouse; R, rabbit.

^a^Dilution used for double immunofluorescence staining.

### Double immunohistochemical labeling

Two-color dual antigen immunostaining was performed to visualize the striatal interneurons (ChAT, SS, PV and CR) together with the striosomal compartment labeled with MOR antibody. We used the protocol previously described in which two sequential immunohistochemistry for light microscopy was performed [28]. Sections were first incubated for 24 h at RT with one of the following primary antibodies: goat polyclonal anti-choline acetyltransferase (ChAT, 1:750); goat polyclonal anti-somatostatin (SS, 1:5,000); goat polyclonal anti-calretinin (CR, 1:10,000); or mouse monoclonal anti-parvalbumin (PV, 1:5,000) (Table 1). After washing, the sections were further incubated for 1 h at RT in the appropriate biotinylated-conjugated secondary antibody (horse anti-goat IgG or goat anti-mouse IgG; 1:500; Vector Laboratories, Burlingame, CA, USA) followed by extravidin-HRP (1:2,000, Sigma-Aldrich, St. Louis, MO, USA). The staining was developed with 3,3’-diaminobenzidine (DAB; 0.05%) and enhanced with nickel ammonium sulfate (0.08%), yielding a dark purplish color. Then, a second staining for 48 h at RT with anti-MOR (1:50,000) was performed, and processed with goat anti-rabbit IgG (1:500; Vector Laboratories, Burlingame, CA, USA), extravidin-HRP (1:2,000) and DAB alone, yielding a brown color.

Double immunofluorescence labeling for MOR and ChAT, SS, PV or CR was performed using green and red fluorescent staining, respectively. Sections were incubated for 48 h at RT with anti-MOR (1:2,000) followed by incubation for 24 h with anti-ChAT (1:500), anti-SS (1:5,000), anti-PV (1:5,000) or anti-CR (1:10,000) (Table 1). Then, the sections were incubated for 1 h at RT in a mixture of Alexa 488 donkey anti-rabbit IgG and Alexa 568 horse anti-goat IgG or Alexa 568 donkey anti-mouse IgG (1:1,000; Thermo Fisher, Waltham, MA, USA). At the end of the staining, the sections were counterstained with 4’,6- diamidino-2-phenylindole (DAPI, 1:250; Molecular Probes, Eugene, OR, USA) and observed with a Leica SP8 laser confocal microscopy (Leica, Wetzlar, Germany).

### Semi-quantitative analysis

Semi-quantitative analysis of optical density (OD) of MOR, TH, DAT and VMAT-2 immunoreactivity (IR) through the rostro-caudal axis (eight levels from Bregma +1.96 mm to -0.56 mm) was performed as described elsewhere using the image analyzing system NIH ImageJ 1.48v (http://rsb.info.nih.gov/nih-image/) [27,29]. The measures were performed from grayscale photomicrographs obtained with a digital camera (VC50) coupled to an optical microscope (Olympus VS120) (10x or 100x objectives). OD values were corrected with the OD from an immunonegative area. We have previously demonstrated that this semi-quantitative methods is suitable to analyse changes in the immunolabeling of a marker under an experimental condition [29]. The percentage of striosomal ablation was calculated as the difference between the OD of MOR IR in the vehicle- and DS-injected hemispheres. The measure of TH, DAT and VMAT-2 IR in the striosomes and matrix was performed using two consecutive MOR immunolabeled slices (anterior and posterior) which are used as a template to identify these two compartments in each section analyzed (Fig 1B) [30,31]. In the case of SNc, OD of TH IR was measured in individual dopamine neurons.

The number of interneurons per mm^2^ (ChAT, SS, PV and CR) was counted and compared between the unlesioned and lesioned hemispheres. Semi-quantitative analysis was also performed to determine the OD of each individual interneuron. This analysis was carry out through the rostro-caudal axis in the lateral and medial part of the CPu.

In all cases, data were expressed as mean percentage OD of control (mean ± SEM).

### Statistical analysis

Statistical analysis was made with Kolmogorov-Smirnov test, Student’s t-tests or Mann-Whitney U test when appropriate. Data were analyzed using SIGMASTAT 2.03 software. Statistical significance was set at P < 0.05.

## Results

### Intrastriatal dermorphin-saporin infusion induces striosomes ablation

**Fig 2:**
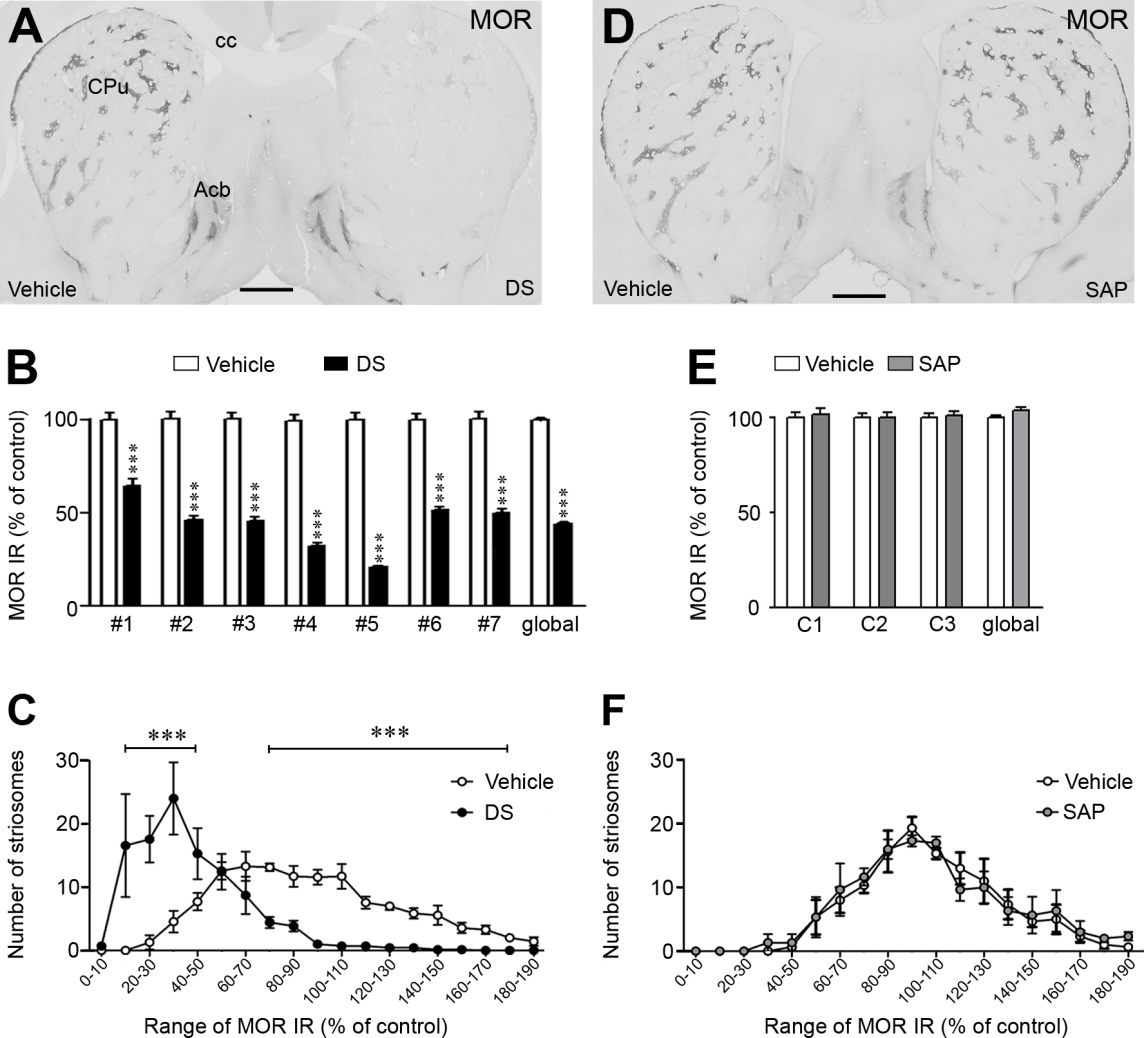
Unilateral dermorphin-SAP intrastriatal injections produced striosomes ablation. (**A and D**) Representative photomicrographs showing the effect of unilateral intrastriatal injections of DS (**A**) and SAP (**D**) on MOR IR. Vehicle injected hemisphere was used as control. (**B and E**) Graphs represent optical density values of MOR IR in the striosomes after DS (#1 to #7; **B**) and SAP (C1 to C3; **E**) injection in each rat. Data represent mean ± SEM and are expressed as percentage of vehicle-injected hemisphere. (**C and F**) Analysis of the number of striosomes in categories ranked by percentages of MOR IR in the rats injected with DS (**C**) or SAP (**F**). Data represent mean ± SEM. Statistical analyses of the data were performed with Mann Whitney U test, ***P < 0.001. Scale bars are 1 mm. Abbreviations: Acb: nucleus accumbens; cc: corpus callosum; CPu: caudate putamen; DS: dermorphin-SAP; MOR: μ opioid receptor; SAP: saporin.

Unilateral injection of DS at two different levels of the rostro-caudal axis of the CPu induced a severe depletion of MOR IR in the striosomes and the subcallosal streak compared with the vehicle-injected hemisphere (Fig 2A). The percentage of decreased MOR IR ranged between 50–80% (Fig 2B), except in two rats in which striosomal ablation was lower than 40% and consequently these animals were discarded from the study. No changes in MOR IR were observed in the nucleus accumbens (vehicle: 100% ± 4; DS: 107% ± 4, Mann-Whitney U test = 8747.5, P = 0.162, n.s.). In control animals (n = 3), SAP injections did not produce changes in MOR IR of the CPu (Fig 2D and 2E).

In the control hemisphere, the frequency of striosomes in categories of MOR IR levels fitted a normal distribution (Kolmogorov-Smirnov test, KS = 0.158, P = 0.142). However, in the lesioned hemisphere, a dramatical displacement of the striosomes frequency toward categories of lower MOR IR was observed (Fig 2C). No differences were found between the SAP- and the vehicle-injected hemispheres (Fig 2F).

**Fig 3:**
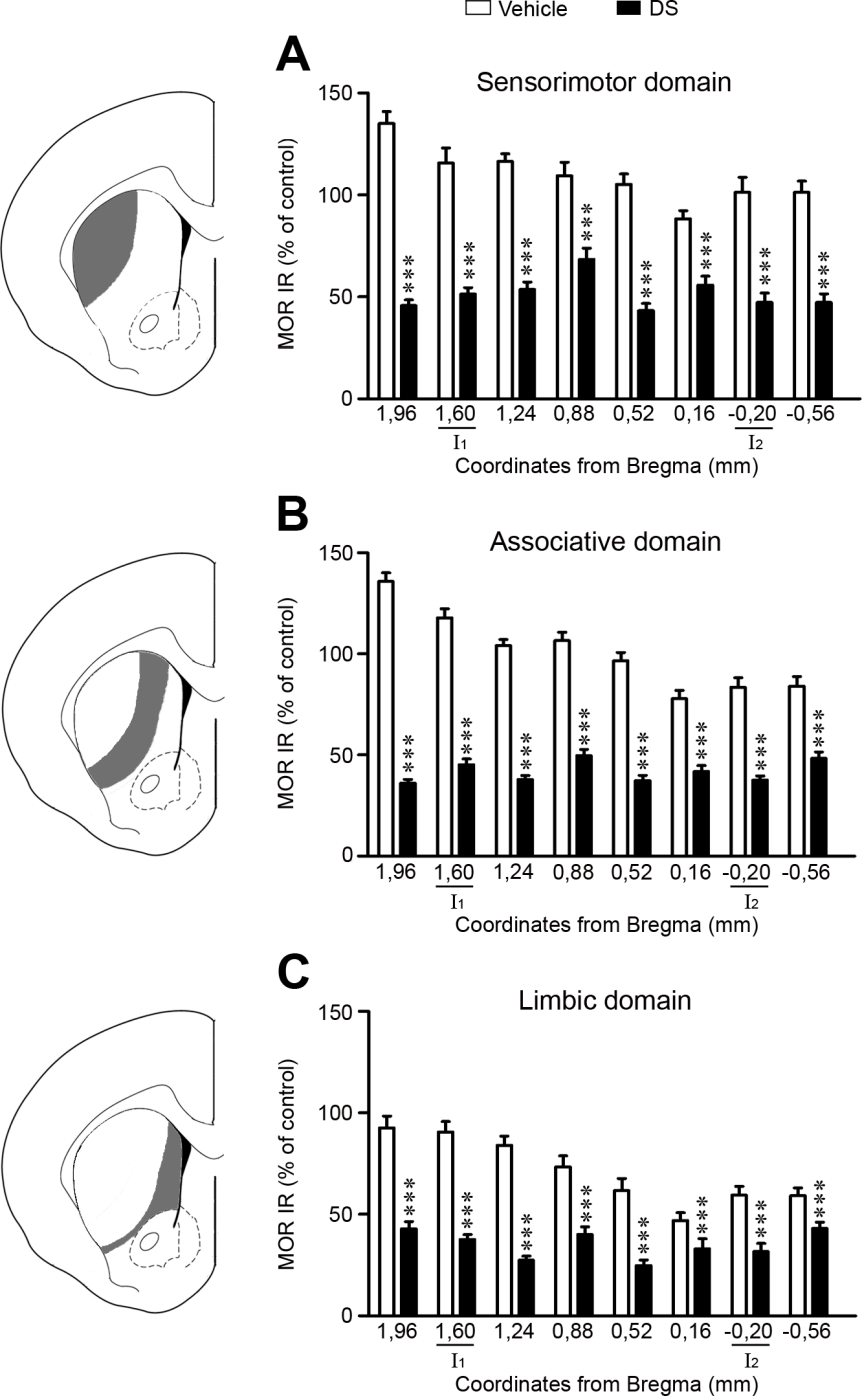
Dermorphin-SAP significantly reduced MOR immunoreactivity through the rostro-caudal axis of the caudate putamen. Graphs represent the optical density values of MOR IR in eight consecutive coronal sections through the rostro-caudal axis of each functional domain of the rat striatum (**A:** sensorimotor; **B:** associative; **C:** limbic). I_1_ and I_2_ indicate the anteroposterior Bregma levels of the injection sites. Data represent mean ± SEM and are expressed as percentage of vehicle treated hemisphere. Statistical analysis of the data was performed with Mann Whitney U test, ***P<0.001.

In order to evaluate the spread of striosomal ablation induced by DS, MOR IR was analyzed in the three functional domains of the CPu, i.e., the sensorimotor (SS), associative (AS) and limbic (L) domains through the rostro-caudal axis. DS significantly decreased MOR IR in the three domains, although it was more evident at the rostral (% ablation: SS, 75–90%; AS, 95–110%; L, 70–80%) than in the caudal levels (% ablation: SS, 45–50%; AS, 30–45%; L, 10–30%) (Fig 3).

### Dermorphin-saporin reduces the number of ChAT and SS interneurons

**Fig 4:**
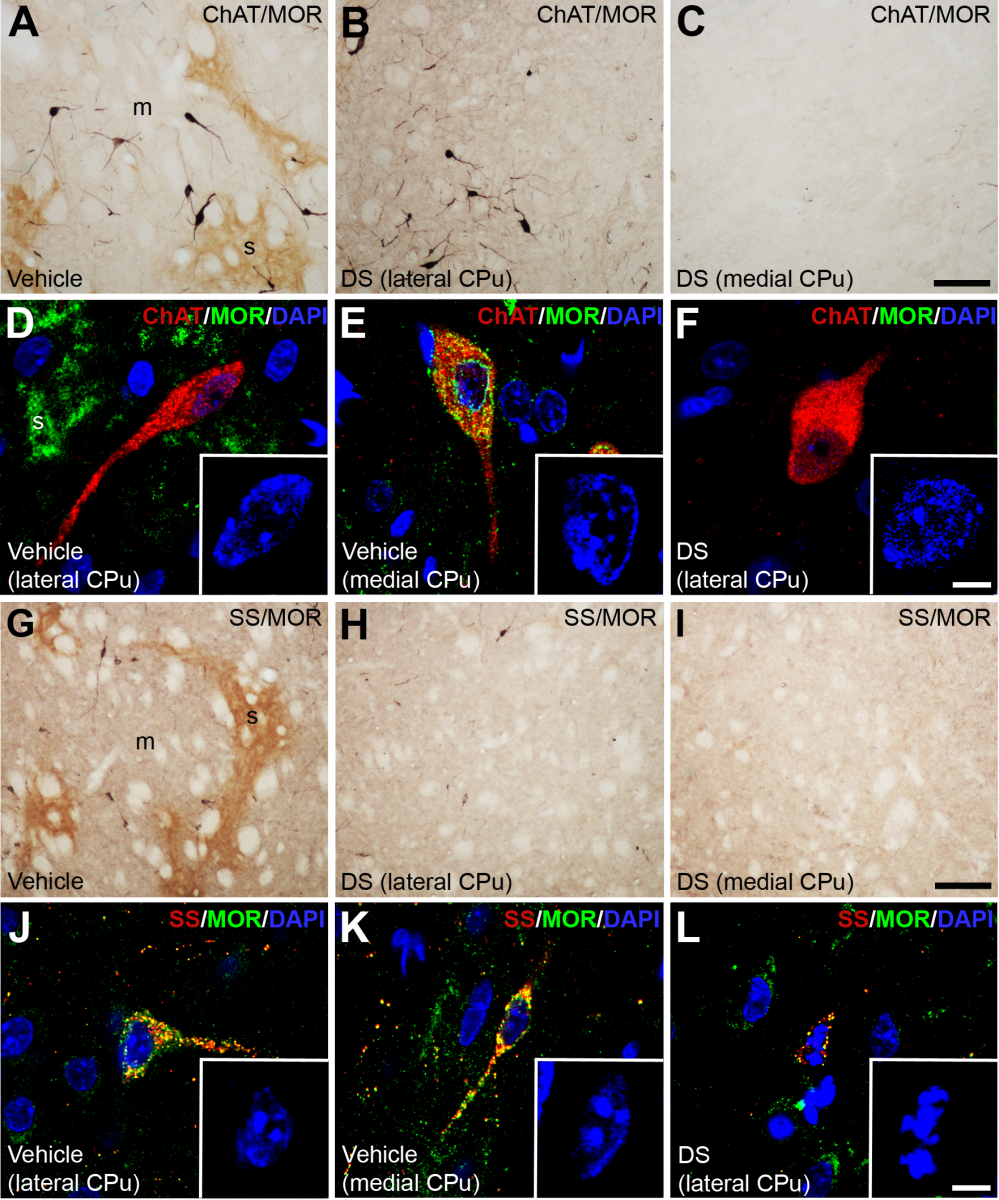
ChAT and SS striatal interneurons are affected by dermorphin-SAP lesion. (**A-C; G-I**) Photomicrographs illustrating by dual labeled immunohistochemistry with anti-MOR (brown) and anti-ChAT (**A-C**) or anti-SS (**G-I**) (dark blue) the impact of DS on these cells in both the lateral (**B-H**) and medial part (**C-I**) of the CPu. (**D-F; J-L**) Confocal laser photomicrographs illustrating the co-localization of MOR (green) with ChAT (**D-F**) and SS (**J-L**) (red). The nuclei are counterstaining with DAPI (blue). Insets show a high magnification of the interneuron nucleus. Abbreviations: CPu: caudate putamen; DS: dermorphin-SAP; m: matrix; s: striosome. Scale bar is 50 μm in A-C, G-I; 10 μm in D-F, J-L; 5 μm in the insets.

In order to evaluate whether DS had an effect on striatal interneurons, four markers were used for their identification, i.e., ChAT, SS, CR and PV. In the medial part of the CPu, ChAT interneurons completely disappeared (Fig 4A and 4C), but remained intact in the lateral part (number, levels of ChAT IR and nuclear morphology) (Fig 4A, 4B and 4F; Table 2). In the case of SS interneurons, DS also reduced the number of these cells in the CPu. A severe depletion of SS interneurons was observed in the lateral part (>75%) (Fig 4G and 4H; Table 2). The remaining SS cells displayed lower levels of IR and a condensed nucleus (Fig 4L; Table 2). A total removal of SS interneurons occurred in the medial part (Fig 4G and 4I; Table 2).

**Table 2:** Effect of striatal DS injection on the interneurons of the rat caudate putamen.

**cells/mm**^**2**^	**Lateral CPu**		**Medial CPu**	
	**Vehicle**	**DS**	**Vehicle**	**DS**
**ChAT**	19.0 ± 2.5	13.1 ± 2.9	26.4 ± 1.9	0
**SS**	13.2 ± 1.2	2.9 ± 1.1***	12.5 ± 1.2	0
**PV**	23.2 ± 0.9	22.2 ± 1.3	11.7 ± 1.6	11.0 ± 2.3
**CR**	10.5 ± 2.4	9.6 ± 1.71	20.3 ± 2.7	20.7 ± 3.1
**OD (% of control)**	**Lateral CPu**		**Medial CPu**	
	**Vehicle**	**DS**	**Vehicle**	**DS**
**ChAT**	100 ± 1.5	100 ± 1.2	100 ± 1.7	-
**SS**	100 ± 2.4	75 ± 2.4***	100 ± 2.3	-
**PV**	100 ± 1.8	100 ± 2.7	106 ± 2.6	107 ± 3.1
**CR**	100 ± 2.8	100 ± 3.3	100 ± 2.0	98 ± 2.1

Data represent the number of cells/mm^2^ and the optical density value (OD) of ChAT, SS, PV and CR immunoreactivity (mean ± SEM). Statistical analysis was performed by Student t’ test

*** P < 0.001 DS vs. vehicle.

**Fig 5:**
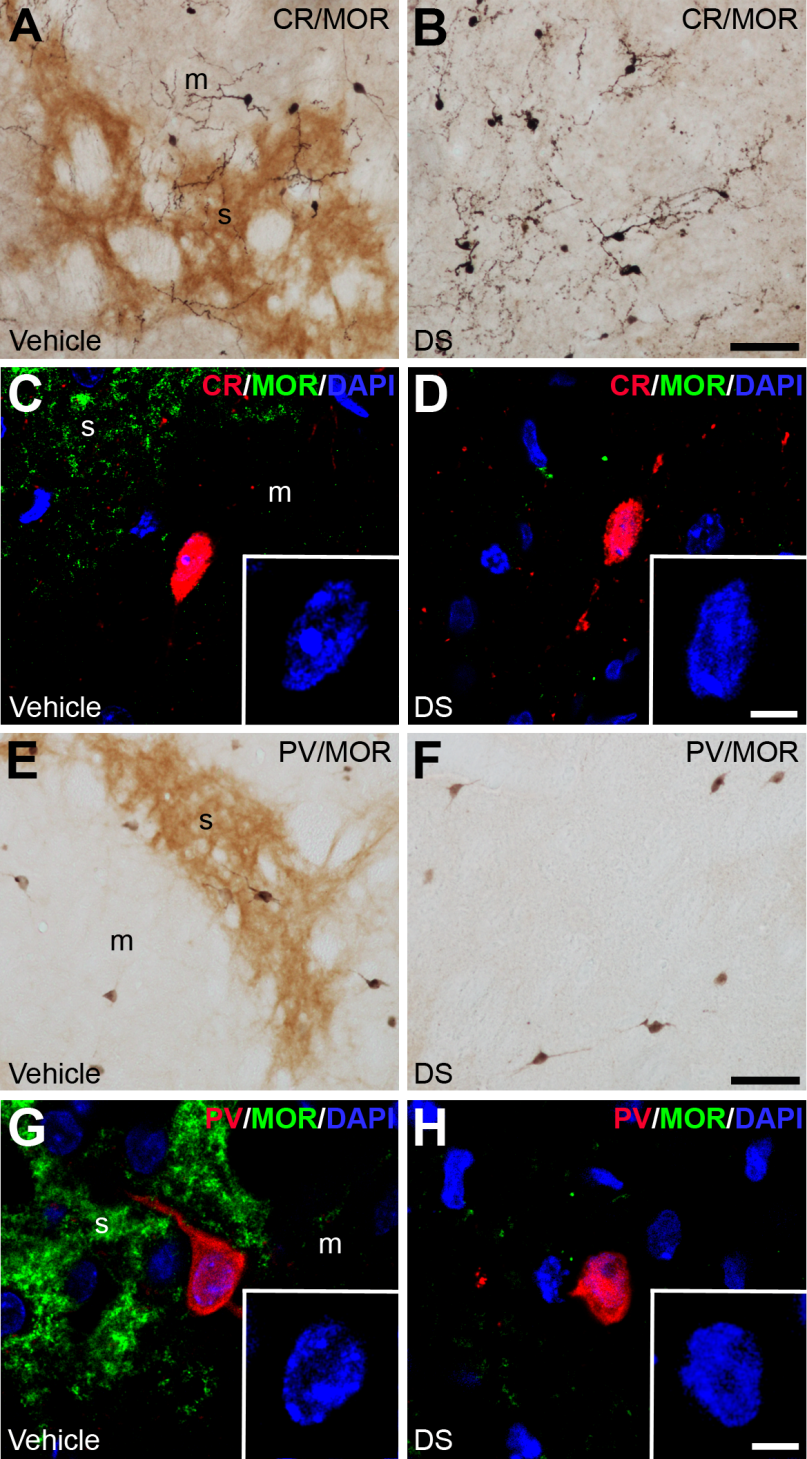
CR and PV striatal interneurons are not affected by dermorphin-SAP lesion. (**A-B; E-F**) Dual labeled immunohistochemistry with anti-MOR (brown) and anti-CR (**A-B**) or anti-PV (**E-F**) (dark blue) to demonstrate the presence of these cells in the vehicle and DS injected hemisphere. (**C-D; G-H**) Confocal laser photomicrographs illustrating the co-localization of MOR (green) with CR (**C-D**) and PV (**G-H**) (red). The nuclei are counterstaining with DAPI (blue). Insets show a high magnification of the interneuron nucleus. Abbreviations: DS: dermorphin-SAP; m: matrix; s: striosome. Scale bar is 50 μm in A-B, E-F; 10 μm in C-D, G-H; 5 μm in the insets.

CR (Fig 5A and 5B) and PV (Fig 5E and 5F) interneurons were not affected by DS injection. In fact, the number and the IR levels of CR and PV interneurons in both the lateral and medial part of the CPu showed no significant differences between the DS and vehicle injected hemispheres (Table 2). Besides, the nucleus of the CR and PV interneurons of the lesioned side displayed the same morphological features than those of the unlesioned hemisphere (insets in Fig 5C, 5D, 5G and 5H).

None of the interneurons in the nucleus accumbens and the cerebral cortex were altered by intrastriatal DS injection.

Because the effect of DS over the striatal interneurons depend on the presence of MOR in these cells, a double immunofluorescence staining was performed. We found that ChAT interneurons of the medial part (Fig 4E) and all SS interneurons (Fig 4J–4L) of the CPu expressed MOR. On the contrary, MOR was not present either in ChAT interneurons of the lateral part (Fig 4D and 4F), nor in CR (Fig 5C and 5D) and PV (Fig 5G and 5H) cells.

### Intrastriatal dermorphin-saporin treatment alter nigrostriatal dopamine pathway

**Fig 6:**
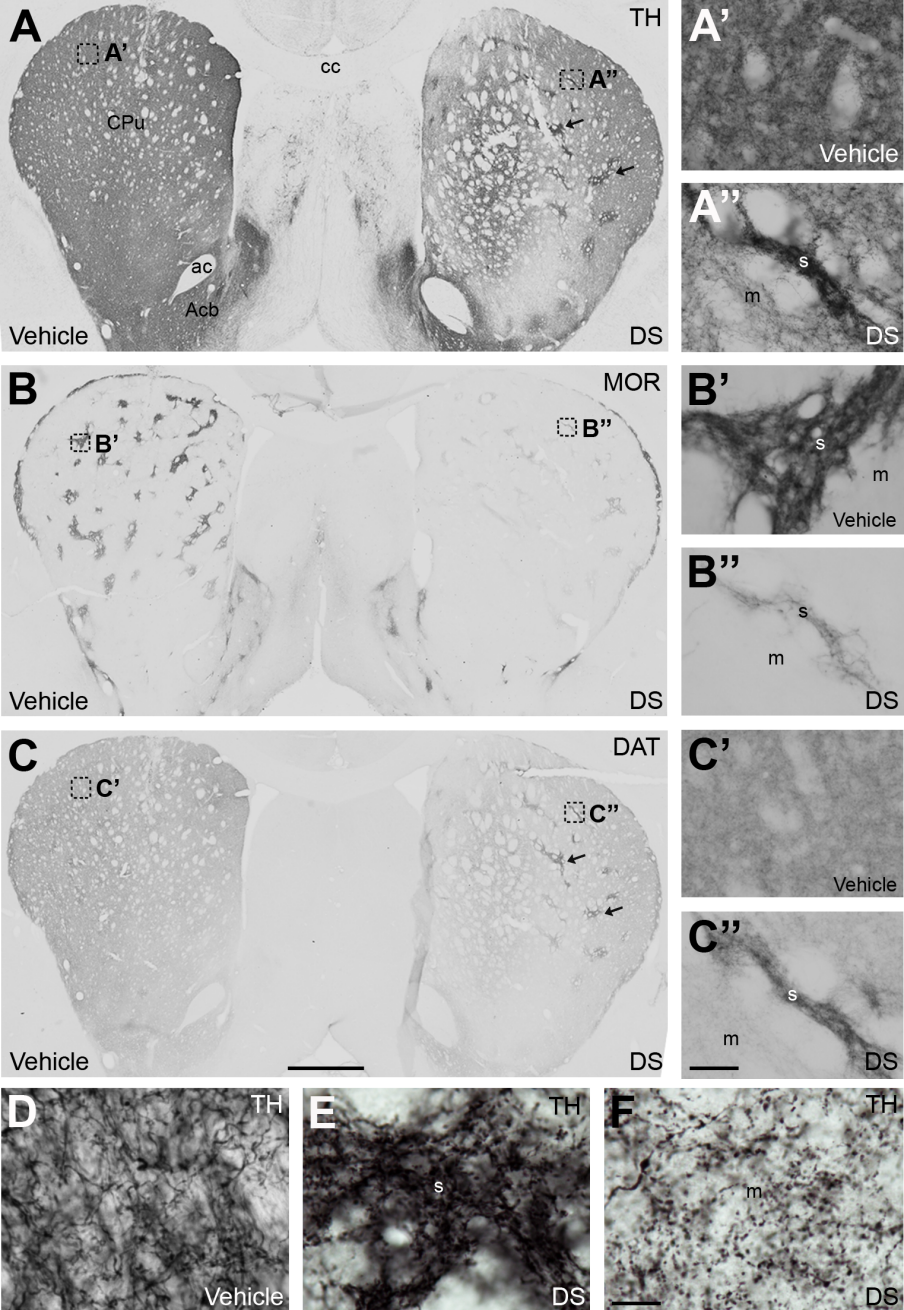
Striosomal ablation affected nigrostriatal dopamine innervation. (**A-C**) Representative photomicrographs illustrating the immunoreactivity of TH (**A**), MOR (**B**) and DAT (**C**) in consecutive sections from rats with unilateral ablation of striosomes. Arrows indicate patches with an enrichment in TH IR and DAT IR in the lesioned hemisphere. (**A’-A”, C’-C”**) High magnification photomicrographs taken from the areas of the CPu pointed as boxes in (**A**) and (**C**) in the unlesioned and lesioned hemispheres. These areas are identified as striosomes in the MOR immunolabeling section (**B’-B”**). (**D-F**) Detailed photomicrographs showing TH IR in the CPu of vehicle (**D**) and DS injected (**E** and **F**) hemispheres. An increase in the size of TH IR varicosities in the striosomes (**E**) and matrix (**F**) compartments is observed in the DS lesioned CPu. Abbreviations: ac: anterior commissure; Acb: nucleus accumbens; cc: corpus callosum; CPu: caudate putamen; DS: dermorphin-SAP; m, matrix; s, striosome. Scale in **C** bar is 1 mm (applies to **A**, **B**, **C**), 50 μm in **C”** (applies to **A’-C”**) and 50 μm in **D** (applies to **D-F**).

To study the effect of the striosomal ablation on the nigrostriatal dopamine pathway, TH, DAT and VMAT-2 have been used as markers of the dopaminergic innervation. MOR immunolabeling in adjacent sections were performed to identify striosomes and matrix compartments (Fig 6B).

**Fig 7:**
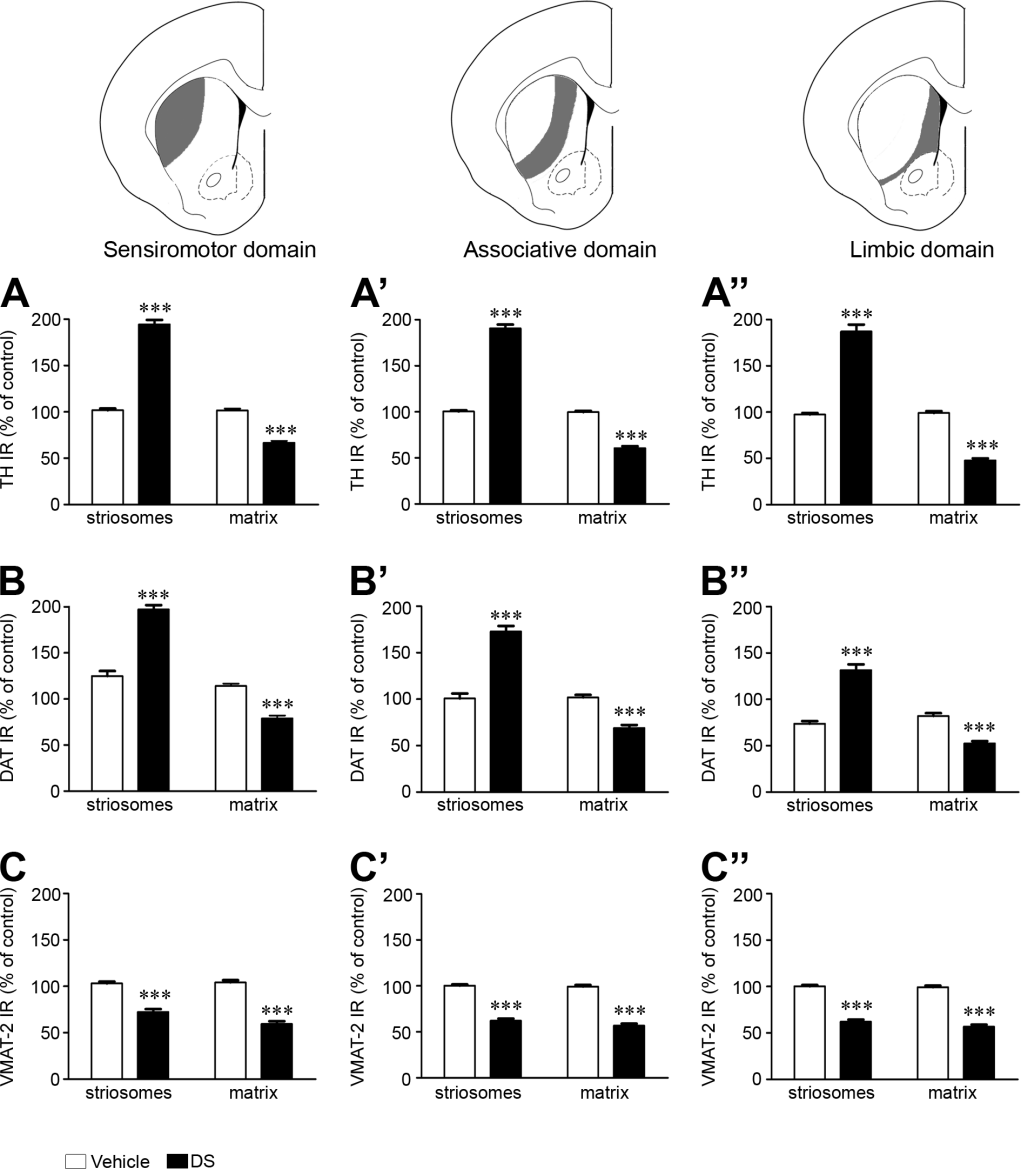
Changes in the nigrostriatal dopamine pathway after striosomes ablation. Graphs represent the semi-quantitative analysis of TH IR (**A-A”**), DAT IR (**B-B”**) and VMAT-2 (**C-C”**) in the striosomes and matrix of vehicle and DS injected hemispheres. The analysis is shown in the three functional domains of the rat CPu (sensorimotor: A, B, C; associative: A’, B’, C’; limbic: A”, B”, C”). Data represent mean ± SEM and are expressed as percentage of vehicle treated hemisphere. Statistical analysis was performed with Student’s t test; *** P < 0.001 DS vs. vehicle.

A homogeneous distribution of immunoreactivity for TH (Figs 6A and 7A–7A”), DAT (Figs 6C and 7B–7B”) and VMAT-2 (Fig 7C–7C”) was observed in the vehicle-injected CPu. However, in the DS lesioned side, changes in the distribution and/or levels of IR for these three markers were observed. A patched pattern of labeling resulted for both TH (Fig 6A–6A”) and DAT (Fig 6C–6C”), which were found to correlate with partial ablated striosomes identified in adjacent sections by immunohistochemistry for MOR (Fig 6B–6B”). In the case of VMAT-2 IR, a homogeneous distribution persisted in the lesioned side, although a decrease of the IR was observed. Semi-quantitative analysis of TH, DAT and VMAT-2 IR was performed in the three functional domains of the CPu. A significant rise of TH IR (SS: 94%; AS: 90%; L: 87%; Fig 7A–7A”) and DAT IR (SS: 73%; AS: 72%; L: 58%; Fig 7B–7B”) and a decrease of VMAT-2 IR (SS: 30%; AS: 40%; L: 40%; Fig 7C–7C”) was observed in the ablated striosomes. In the matrix, a decrease of TH IR (SS: 44%; AS: 40%; L: 48%; Fig 7A–7A”), DAT IR (SS: 36%; AS: 31%; L: 30%; Fig 7B–7B”) and VMAT-2 IR (SS: 40%; AS: 44%; L: 50%; Fig 7C–7C”) was evident. It was noted that the increase or decrease of TH IR was associated with a higher or smaller amount of TH IR processes, respectively. Besides, a swelling of TH IR varicosities in the whole lesioned CPu was observed (Fig 6D–6F).

**Fig 8:**
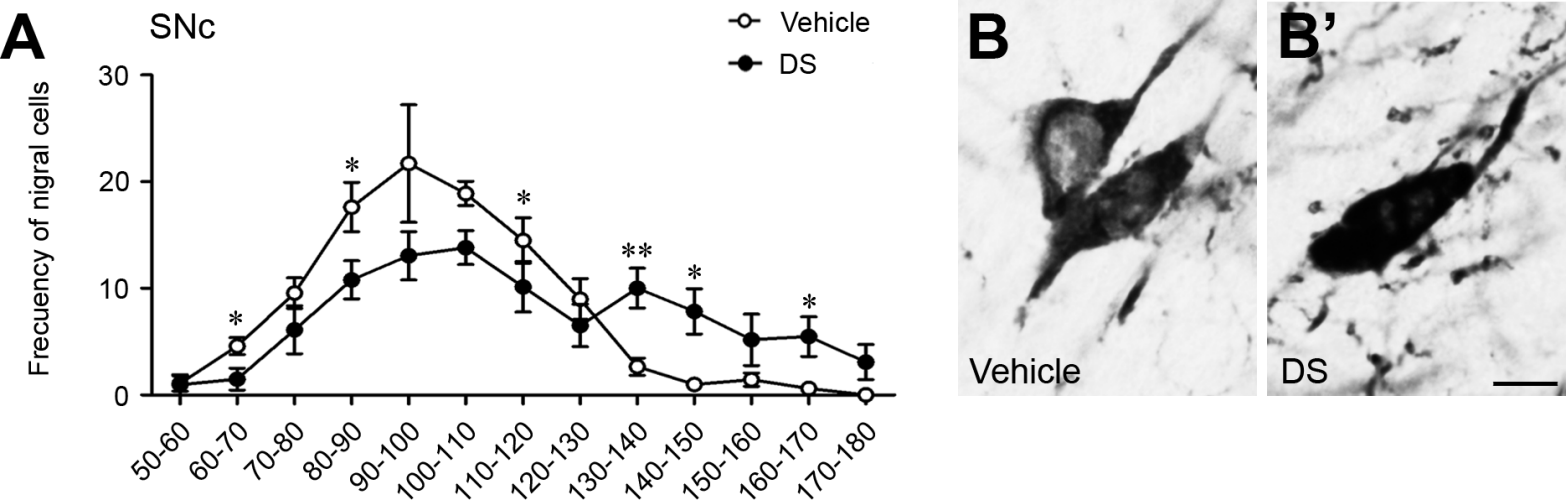
Striosomal ablation affected a subset of nigral dopamine neurons. (**A**) Frequency of nigral cells (expressed as percentage of total cells analyzed) in categories ranked by percentages of TH IR in the control and lesioned hemispheres. Statistical analyses of the data were performed with Student’s t test, *P<0.05, **P<0.01. (**B** and **B’**) High magnification photomicrographs showing TH IR cells of the SNc. Scale bar is 5 μm.

The lesion of the striosomes produced the rise of TH IR (by 50%) in a subset of nigral dopamine neurons (32% of total), whereas the remaining cells (68% of total) showed TH IR levels similar to those observed in the control hemisphere (Fig 8).

## Discussion

The functions mediated by the striosomes of the CPu are not completely understood, although data collected in the last few decades suggest that they exert a limbic control over the surrounding sensorimotor and associative matrix [7]. Single-cell and tract-tracing experiments have revealed that striosomes directly projects to the dopaminergic neurons of the SNc [12,13,32]. The goal of the current study was to analyze the role of the striosomes on the regulation of nigrostriatal dopamine pathway. In order to examine this question, we have induced selective ablation of the striosomes by intrastriatal infusion of the neurotoxin DS.

DS was used for the first time to induce the deficit of brainstem medullary cells which express MOR [22]. Later on, this methodology was also applied to induce the degeneration of the striosomes in the mouse and rat CPu [21,23–25]. These studies showed that a unique injection of DS into the CPu induced an incomplete depletion of the striosomes, since it was restricted around the injection site. As the CPu is a large nucleus, it is conceivable that DS is not able to reach all the striosomes along the rostro-caudal axis. In order to induce a maximal neuronal degeneration of the striosomes, we have improved this method performing the infusion of DS at two different sites of the rostro-caudal axis. We have systematically tested the effect of two-sites infusion of DS and we have demonstrated a severe depletion of the striosomes in the whole CPu.

It should be noted that the ablation of the striosomes of the rostral CPu is greater than in the caudal part. This result supports the newly emerging understanding of the nonuniform characteristics of the striosomal compartment, which is manifested by a biochemical composition diversity [6]. For example, a rostro-caudal MOR density gradient has been well documented, with higher expression of this receptor in the striosomes of the rostral CPu [33] (own observation). Therefore, the striosomal neurons of the caudal levels, with fewer MOR, would be less affected by the DS neurotoxic. The different vulnerability of the striosomes to DS could also be explained by the presence of calbindin in some striosomes, specifically in those of the ventro-medial CPu [34]. It is known that calbindin has a protective role preventing cell degeneration as a result of its ability to bind calcium [35].

It is well known that MOR is mainly expressed by the MSNs of the striosomes [36–38]. However, there is also evidence of its presence in ChAT interneurons located in the limbic/prefrontal territory of the CPu [39–41], whereas it seems to be absent from those in the sensorimotor division [39]. Here, we provide unequivocal evidences for the exclusive presence of MOR in a subset of ChAT interneurons located in the medial division of CPu, since double immunolabeling experiments demonstrate that cholinergic interneurons of the lateral CPu are devoid of MOR. These cholinergic interneurons of the lateral CPu display identical characteristics (number, ChAT IR and nuclear morphological) than those in the control hemisphere [42]. These results corroborate that these cells are not affected by DS lesion. The discrimination of two subpopulations of cholinergic interneurons, according to their content in MOR, could clearly explain our observation of a complete disappearance of ChAT cells exclusively in the associative and limbic domains. Recently, it has been demonstrated that ChAT interneurons of the lateral striatum are involved in the development of some of the symptoms of Tourette syndrome [42], supporting the concept of different subpopulations of ChAT cells.

In addition, we report for the first time that among the striatal GABAergic interneurons, only SS cells express MOR. The present findings support our observation that PV and CR interneurons were not affected by DS, whereas SS interneurons almost disappeared. In this view, the few remaining SS cells in the lesioned hemisphere showed condensed nuclei, which are characteristic of cells undergoing degeneration [43].

**Fig 9:**
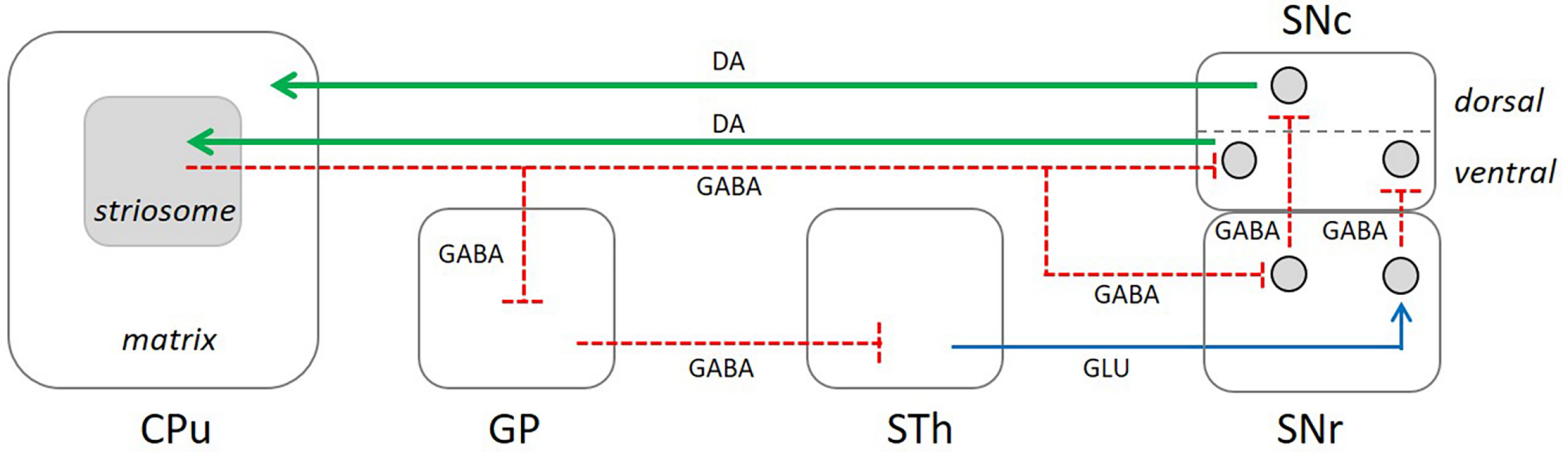
Schematic diagram of striosomal circuits into the basal ganglia. In the striatum the striosomal and matrix compartments are depicted whereas in the SNc the dorsal and ventral tiers are illustrated. Red lines indicate the direct GABAergic projections from the striosomes to the SNc and their collaterals to the GP and SNr. Green lines point the dopaminergic projections from the SNc to the striosomes and matrix. Abbreviations: SNc: substantia nigra pars compacta; SNr: substantia nigra pars reticulata; GP: globus pallidus; STh: subthalamic nucleus; DA: dopamine; Glu: glutamate.

The ablation of the striosomal compartment produces a dramatic deregulation of the dopamine nigrostriatal pathway, with an increase of dopamine signaling in the striosomes and a decrease in the matrix. This observation is consistent with previous studies that have demonstrated the critical role of MOR in the regulation of striatal dopamine release [16]. The increase of TH IR in the striosomes could be the consequence of MSNs degeneration in this compartment and, therefore, the disappearance of their inhibitory action onto the dopaminergic neurons of the SNc throughout the direct striatonigral projection (Fig 9) [4,11–13,44]. This result is in good correlation with the increase of TH IR in a sub-population of the SNc neurons. A recent report has highlighted that axons of the striosomal MSNs form contacts with the ventrally extending dendrites arising from clusters of dopamine neurons in the ventral tier of the SNc [44]. It has been described that MOR also exists in scattered non-striosomal cells that therefore might be affected by DS treatment [45]. However, despite the localization of these neurons in the matrix compartment, they are similar to striosomal neurons in terms of genetics, neurochemistry, membrane properties, excitability, basal inhibitory synaptic transmission, response to opioid agonists, and functional connectivity [46]. These neurons have been termed as “exo-patch”. Thus, the depletion of “exo-patch” neurons by DS might have the same impact on the dopaminergic neurons of the SNc than the striosomal MSNs.

On the other hand, Matsuda et al. [32] have demonstrated that the axonal arborization of single nigrostriatal neurons located in the ventral tier of the SNc provides dopamine signals sent back with some preference for the striosomes. Thus, it is likely that the striosomes exert a powerful control over a specific cluster of dopaminergic neurons located in the ventral part of the SNc (Fig 9). Additionally, an indirect pathway involving colaterals of the striosomal MSNs to the GP [12] could contribute to the increase of reciprocal dopamine signaling (Fig 9). The rise of DAT and the decrease of VMAT-2 observed in the ablated striosomes could represent a compensatory mechanim in response to the increase of dopamine signaling.

Another consequence of striosomal MSNs depletion was the decrease of dopamine signaling in the matrix, probably due to the loss of colaterals from striosomal MSNs which project to the SNr [12] (Fig 9). In this case, a deregulation of the dopamine neurons located in SNc dorsal tier could occur, since these neurons project back to the matrix [13]. The downregulation of DAT and VMAT-2 in the matrix might be related to the decrease of dopamine innervation, rather than a regulatory mechanism. More studies will be necessary to found out the alterations that striosomes ablation produces in the dopaminergic terminals in the matrix. Besides, the loss of ChAT and SS interneurons, which exert a regulatory control over the MSNs located in both the striosomes and matrix compartments, could additionally contribute to the deregulation of the striato-nigral-striatal loop. In this sense, the physiological responses of MSNs due to changes in striatal cholinergic tone are difficult to evaluate, since its will be conditioned by the type of mediating muscarinic receptor. The MSNs can express: i) the excitatory M1 receptor, which activates G_q/11_ proteins and induces the activation of phospholipase C pathway; ii) the inhibitory M4 receptor, which is coupled to G_i/0_ proteins and decreases activity of adenylyl cyclase; or iii) both M1 and M4 receptors [47–49]. Furthermore, it has been described a predominant expression of M4 receptor in the striosomal than the matrix compartment [50], suggesting different physiologic responses related to distinct striatal circuits.

In summary, the results of the present paper suggest a crucial role of the striosomes in the maintenance of the striatal dopamine tone, since the degeneration of this compartment produces an important imbalance in the dopamine basal ganglia circuits. This is corroborated by the relationship between the striosomes and the correct execution of complex movements [51,52].

Our results also highlight the ablation of striosomes as a powerful tool that can help to understand the complexity of the striatal organization and its relationship with neurological disorders like Huntington’s disease, X-linked dystonia-parkinsonism or Parkinson’s disease [1,7,18,53].

## Supporting information

S1 Data Complete data sets used in this study. (ZIP)
